# Changes in microbiota composition, bile and fatty acid metabolism, in successful faecal microbiota transplantation for *Clostridioides difficile* infection

**DOI:** 10.1186/s12876-018-0860-5

**Published:** 2018-08-28

**Authors:** Jillian R.-M. Brown, Burkhardt Flemer, Susan A. Joyce, Akbar Zulquernain, Donal Sheehan, Fergus Shanahan, Paul W. O’Toole

**Affiliations:** 1 APC Microbiome Institute, University College Cork, National University of Ireland, Cork, Ireland; 2 School of Microbiology, University College Cork, National University of Ireland, Cork, Ireland; 3 School of Biochemistry and Cell Biology, University College Cork, National University of Ireland, Cork, Ireland; 4 Department of Medicine, University College Cork, National University of Ireland, Cork, Ireland

**Keywords:** *Clostridioides difficile*, Faecal microbiota transplantation, Bile acids, Fatty acids, Archaea

## Abstract

**Background:**

Alteration of the gut microbiota by repeated antibiotic treatment increases susceptibility to *Clostridioides difficile* infection. Faecal microbiota transplantation from donors with a normal microbiota effectively treats *C. difficile* infection.

**Methods:**

The study involved 10 patients with recurrent *C. difficile* infection, nine of whom received transplants from individual donors and one who received a donor unit from a stool bank (OpenBiome).

**Results:**

All individuals demonstrated enduring post-transplant resolution of *C. difficile-* associated diarrhoea. Faecal microbiota diversity of recipients significantly increased, and the composition of the microbiota resembled that of the donor. Patients with *C. difficile* infection exhibited significantly lower faecal levels of secondary/ bile acids and higher levels of primary bile acids. Levels of secondary bile acids were restored in all transplant recipients, but to a lower degree with the OpenBiome transplant. The abundance increased of bacterial genera known from previous studies to confer resistance to growth and germination of *C. difficile*. These were significantly negatively associated with primary bile acid levels and positively related with secondary bile acid levels. Although reduced levels of the short chain fatty acids, butyrate, propionate and acetate, have been previously reported, here we report elevations in SCFA, pyruvic and lactic fatty acids, saturated, ω-6, monounsaturated, ω-3 and ω-6 polyunsaturated fatty acids (PUFA) in *C. difficile* infection. This potentially indicates one or a combination of increased dietary FA intake, microbial modification of FAs or epithelial cell damage and inflammatory cell recruitment. No reversion to donor FA profile occurred post-FMT but ω-3 to ω-6 PUFA ratios were altered in the direction of the donor. Archaeal metabolism genes were found in some samples post FMT.

**Conclusion:**

A consistent metabolic signature was identified in the post-transplant microbiota, with reduced primary bile acids and substantial restoration of secondary bile acid production capacity. Total FA levels were unchanged but the ratio of inflammatory to non-inflammatory FAs decreased.

**Electronic supplementary material:**

The online version of this article (10.1186/s12876-018-0860-5) contains supplementary material, which is available to authorized users.

## Background

*Clostridioides difficile* infection is an important hospital acquired infection [1] with 440 cases reported for the last quarter of 2017 in Ireland [2]. *C. difficile* is a Gram-positive anaerobic bacterium which produce spores that are very resistant to chemicals, heat and radiation but which may germinate in the presence of bile salts and glycine into metabolically active toxin-producing cells [3, 4]. *C. difficile* is present in up to 3% of healthy adults, 20% of individuals on antimicrobial therapy and up to 90% of healthy asymptomatic new-born infants. *C. difficile* colonization is inversely correlated with immunoglobulin A levels in infants [5] and IgG in adults [6]. Antibiotic treatment affects the gut microbiota resulting in microbial and metabolic signature alterations which favour *C. difficile* overgrowth [7, 8]. Growth of *C. difficile* is normally suppressed by other anaerobic bacteria, but *C. difficile* colonisation rates increase with age and reduced stability of the gut microbiome [9]. Decreasing *Bacteroidetes* abundance may promote *C. difficile* colonization because it is associated with higher abundance of *Ruminococcus gnavus* and *Clostridium nexile*, both of which produce a trypsin-dependant antimicrobial peptide affecting other clostridial species but not *C. difficile* [10]. *C. difficile* colonization resistance is maintained by a number of key Firmicutes species including short chain fatty acid (SCFA) (butyrate) producers as well as other species from Clostridium clusters IV and XIVa [11]. The microbiome is responsible for modifying diverse bile acids in the small intestine and colon. Members of Clostridium cluster XIVa metabolize free bile acids (Cholic acid (CA) and Chenodeoxycholic acid (CDCA)) to produce signal-altering, hydrophobic bile acids (Deoxycholic acid and Lithocholic acid respectively). Upstream conversions by bacterial bile salt hydrolase enzymes from a wide variety of taxa are the gateway to further modifications. One important factor affecting the growth of *C. difficile* is the generation of secondary bile acids (deoxycholic acid from CA and lithocholic acid from CDCA) by 7-alpha-dehydroxylation [12, 13]. In healthy individuals *C. difficile* appears to be suppressed by secondary bile acid levels in the colon. Interestingly, highly virulent *C. difficile* strains are significantly more tolerant of LCA [14].

The potential to counteract *C. difficile* overgrowth by replenishing microbial diversity in the gut is well established. Nearly 30 years ago, rectal instillation of a mixture of 10 different bacterial species successfully treated relapsing *C. difficile* diarrhoea in 5 patients [15]. Re-colonization and the reintroduction of microbial diversity also have the effect of altering downstream metabolite production. However, while SCFAs in the gut are altered in the murine *C. difficile* infection model, [4, 16] the administration of SCFA alone is insufficient to alleviate disease symptoms [16]. Nevertheless, restoration of the bile acid and salt balance appears to be an effective means by which the gut microbiota suppresses *C. difficile* colonization and outgrowth [17].

Methanogens belong to the domain Archaea, and although variably present in the intestine, their role in human disease is not well established. The predominant methanogen in the human gut is *Methanobrevibacter smithii*, [18–20]. *Methanosphaera stadtmanae* is also found in humans, but less frequently [21]. Some methanogens harbour genes that were possibly acquired by lateral gene transfer from gut *Firmicutes*, allowing their growth in the presence of bile salts [22]. These archaea are capable of symbiotic interactions with other bacteria that enhance SCFA production [23, 24].

The aim of this study was to investigate microbial community dynamics and metabolic changes associated with successful FMT. We used 16S rRNA gene profiling and shotgun metagenome sequencing to compare the microbiota of healthy donors and patients with *C. difficile* infection, before and after FMT. We determined faecal bile and fatty acid profiles to investigate associations with microbiota changes observed.

## Methods

### Patients

All patients were recruited by direct referral from outpatient gastroenterology clinics or the in-patient services at Cork University Hospital, Ireland. Patients had a history of diarrhoea and toxin-positive *C. difficile* infection following antibiotic therapy (Table 1). Inclusion criteria for FMT included recurrent *C. difficile* diarrhoea following primary anti-*C. difficile* therapy. The study was approved by the Clinical Research Ethics Committee of the Cork Teaching Hospitals. Informed written consent was obtained from all patients. A complete antibiotic history was recorded. A faecal sample was collected and tested for *C. difficile*, enteric infections and parasites. Diagnostic *C. difficile* assay was by real time PCR (polymerase chain reaction) for *tcdB* (*C. difficile* toxin B) in faeces (EntericBio®, Serosep Ltd., Limerick, Ireland). Serological screening prior to FMT included Human Immunodeficiency virus, Hepatitis B & C and syphilis.

**Table 1:** Clinical characteristics of the patient population in this study

Patient	Age	Sex	Antibiotics Pre *C. difficile* infection	Antibiotics used to treat *C. difficile* infection	Resolution of *C. difficile* infection
1	35	F	Co-amoxiclavMetronidazole	Metronidazole, Vancomycin, Fidaxomicin	No
3	35	F	As above	As above	Yes
2	24	F	Erythromycin Azithromycin	Vancomycin, Fidaxomicin	Yes
4	54	F	Co-amoxiclav	Metronidazole, Vancomycin, Fidaxomicin	Yes
5	69	F	Tazobactam, Piperacillin Co-amoxiclav	Metronidazole Vancomycin Fidaxomicin	Yes
6	65	F	Flucoloxacillin Calvepen Benzylpenicillin Co-amoxiclav	Metronidazole, Vancomycin	Yes
7	90	F	Cefuroxime Cephalexin	Metronidazole Vancomycin Fidaxomicin	Yes
8	58	F	Tazobactam Piperacillin Vancomycin, Meropenem	Metronidazole Vancomycin Fidaxomicin	Yes
10	22	M	None	Vancomycin	Yes
11	68	M	Tazobactam Piperacillin	Metronidazole Vancomycin Fidaxomicin	Yes

### Donor identification and screening

Patients and relatives were asked to identify potential donors from among their family and friends. The donor’s past and current medical history, including any abdominal symptoms, antibiotic use, travel history and assessment for transmissible disease, were recorded and reviewed. Informed written, donor consent was obtained. Donors underwent laboratory serologic testing for Hepatitis B, C, cytomegalovirus, Epstein Barr virus, Human Immunodeficiency Virus and *Treponema pallidum*. Donor stool was screened for *C. difficile*, common enteropathogens, ova, cysts and parasites as described [25]. In the single case that a suitable personal donor was not available, a banked stool donor unit from Open-Biome (www.openbiome.org) was used.

### Anaerobic preparation of inoculum for faecal microbiota Transplantation (FMT)

Faecal samples were collected from the donor and processed in a dedicated anaerobic cabinet less than 1 h post voiding. Published protocols [26, 27] were used for the preparation of the faecal suspension and infusion, with the modification that all processes were carried out anaerobically under N_2_ gas flow. Briefly, the sample was weighed (> 50 g) and homogenized in 250 ml physiological saline (0.9%). The faecal slurry was then sieved through a sterile 0.25 mm sieve lined with two unfolded gauze sheets to remove larger particles and to prevent syringe blockage. The faecal suspension container was topped up with saline leaving no headspace and immediately transferred anaerobically (or on ice if using Open Biome sample) to the endoscopy suite, where it was administered to the patient within 1 h after preparation.

### FMT procedure

The night before the procedure, the patients took bowel preparation containing polyethylene glycol (Movicol®, Norgine Ltd) to remove residual luminal antibiotics and faecal material. The patients underwent an esophago-gastro-duodenoscopy and full colonoscopy under conscious sedation. Faecal suspension (120 ml) was infused in the distal duodenum via a gastroscope. The remaining 180 ml was administered commencing from the distal ileum and right colon during withdrawal of the colonoscope. Patients were clinically followed and stool was obtained for *C. difficile* toxin 2 weeks and 8 weeks post procedure. Treatment was defined as successful if there was symptomatic clinical improvement and when *C. difficile* testing was negative.

### Sample collection

Faecal samples were collected from patients prior to FMT and between 12 and 180 days following the procedure. An aliquot of donor stool was collected on the day of the procedure. DNA (deoxyribonucleic acid) from stool samples was extracted immediately. Stool from both donor and recipient was stored at − 80 °C for bile and fatty acid analysis.

### DNA extraction, library preparation and 16S amplicon sequencing

Genomic DNA was extracted from faecal samples (0.25 g) using the Repeat Bead Beating (RBB) method of Yu and Morrison [28] with the following modifications. Sterile zirconia beads (0.5 g) collections of (one 3.0 mm bead, 0.1 g of 0.5 mm beads, and 0.3 g of 0.1 mm beads) were used. Faecal samples were homogenised via bead beating for 90 s (Mini-Beadbeater™, BioSpec Products, Bartlesville, OK, USA), with the samples cooled on ice for 60 s before another 90 s bead beating. Pooled supernatants were incubated with 350 ml of 7.5 M ammonium acetate (Sigma). The extraction then proceeded as per the RBB extraction protocol. Genomic DNA was visualised on 1% agarose gel and quantified using the Nanodrop 1000 (Thermo Scientific, Ireland). Extracted genomic DNA was stored at − 20 °C until amplification. Bacterial primers used for PCR amplification targeting the V3-V4 hypervariable region of the 16S rRNA gene are listed in Additional file 1: Table S1 [29]. Illumina overhang adapter sequences were appended to the 16S rRNA gene specific primer sequence. The PCR master mix comprised 0.2 μM forward and reverse 16S amplicon primers (EUROFINS, Ebersberg, Germany), 15 μl of 1X Phusion Taq High-Fidelity Mix (Thermo Scientific, Ireland), 10 ng DNA and nuclease free water to 30 μl. The following PCR program was used: 1. 98 °C for 30s, 2. 98 °C for 10s, 3. 55 °C for 15 s, 4. 72 °C for 20s, 5. 72 °C for 5 min. Steps 2-4 were repeated for 25 cycles. Amplicons were visually checked on 2% agarose gels and purified by magnetic purification using SPRI select beads (Beckman Coulter). The amplicon was then bar-coded using a limited cycle PCR and Nextera XT dual index barcodes (Illumina, Netherlands) (Additional file 1: Table S1). The PCR master mix comprised 5 μl forward and reverse Nextera primer respectively (Illumina, Netherlands), 25 μl of 1X Phusion Taq High-Fidelity Mix (Thermo Scientific, Ireland), 5 μl PCR amplicon and nuclease free water to 50 μl. The PCR program was the same as the aforementioned with the exception of 8 cycles repeated for steps 2-4. Amplicons were visually checked on 2% agarose gels, purified using SPRI select beads (Beckman Coulter) and quantified through fluorometric Qubit dsDNA HS Assay Kit (Thermo Scientific, Ireland). Equimolar amounts of DNA per amplicon were then pooled and sequenced by GATC Biotech, Germany on an Illumina MiSeq using a 2 × 250 bp paired end sequencing run.

### Shotgun metagenomic sequencing

Genomic DNA was extracted as described above. The DNA quality was checked on 1% agarose gel as well as a Bioanalyser. Five μg of high molecular weight DNA were sent to BGI TECH Solutions CO (Hong Kong) for sequencing on Illumina HiSeq (HiSeq 2500/4000) using 2 × 125 bp paired-end chemistry.

### Bile and fatty acid measurements

Bile acids were extracted as previously described [30]. Briefly, 100 mg faeces was added to Dyna beads® (Roche) with 300 μl of ice cold 50% methanol containing deuterated internal standards of both CA and CDCA, then subjected to five 30 s rounds of extraction in a Dynalyser machine (Roche) at 6000 rpm. The mixture was vortexed and then centrifuged for 10 mins at 10,000 g and the supernatant transferred to a fresh tube. Two ml of ice cold acetonitrile (ACN) with formic acid was added to each tube, vortexed and agitated at room temperature for 1 h. Samples were centrifuged again to pellet the debris and the supernatant was added to fresh tubes containing 1 ml of ice cold 100% ACN. The samples were vortexed and dried under vacuum at 4 °C. The dried extracted acids were re-suspended in 150 ml of ice cold 50% methanol.

Conjugated bile salts and free bile acids for standards were purchased from Sigma Aldrich and Steraloids, Inc. (Wicklow, Ireland and Newport, Rhode Island). HPLC-grade methanol, acetonitrile, water, ammonium acetate, ammonium formate, ammonium hydroxide, formic acid, and acetic acid and water were obtained from Fisher Scientific (Fair Lawn, NJ). Deuterated cholic acid (D-2452) and deuterated chenodeoxycholic acid (D-2772) were purchased from CDN Isotopes Inc. Standards were prepared as 1 mg/ml stock solutions of individual sulphated bile acids in water: methanol (1:1). They were subsequently combined to a final volume of 1.0 ml in water to give a stock concentration of 40 mg/ml. Subsequent dilutions were made to which the same volume of deuterated standards was added. Fatty acids were treated similarly but resuspended in 100% methanol. These standards were utilized to create standard curves for each analyte examined (Additional file 1: Tables S2 and S4).

### Ultra Performance Liquid Chromatography Tandem Mass Spectrometry (UPLC-MS)

UPLC-MS was performed as described [30] with minor modifications. Briefly, 5 μL from each sample were injected onto a C18 Acquity column (Waters Corp. Herts, United Kingdom). Each sample was run in triplicate. Extracts were eluted using a 25-min gradient of 100% A to 100% B (A, water, 0.1% formic acid; B, acetonitrile, 0.1% formic acid) at a flow rate of 500 μL/min and column temperature of 40 °C. Samples were analyzed using an Acquity system (Waters Ltd. Herts, United Kingdom) coupled online to an LCT Premier mass spectrometer (Waters MS Technologies, Ltd.) in negative electrospray mode with a scan range of 50–1000 m/z. Bile acids, long and medium chain fatty acids ionize strongly in negative mode, producing a prominent [M-H] − ion. Capillary voltage was 2.4 Kv, the sample cone was 35 V, the desolvation temperature was 350 °C, the source temperature was 120 °C, and the desolvation gas flow was 900 L/h. Analysis was performed using Waters software Targetlynx for exact quantification against a standard curve and Markerlynx for non-biased principle component analysis UPLC.

### Bioinformatic and statistical analysis

16S amplicon sequences were processed as described elsewhere [31]. Shotgun sequencing data was processed as follows: Sequencing reads were aligned to the human genome (hg20) using bowtie2 [32] and filtered for quality using trimmomatic [33]. Filtered reads were then analysed using humann2 [34].

Statistical analysis was carried out using the programming language R (Additional file 1: Table S5) [35]. Standard visualizations were carried out using base R or ggplot2 [36]. Unweighted UniFrac distances were calculated in QIIME [37] using data rarified to the lowest sequencing depth per sample (4211 sequences) and were visualized using function s.class [38]. Statistical significance was established using permutational analysis of variance (PERMANOVA) using distance matrices and the function adonis of the vegan package [39]. Statistical significance for α-diversity measures was established using a two-tailed Student’s t-test. Normal distribution of α-diversity values was confirmed using a Kolmogorov-Smirnov test (all *p-*values > 0.05). Differential abundance of microbial genera between groups was assessed using DESeq2 [40] with an FDR < 0.1. Other *P*-values were adjusted using the function p.adjust (stats package of base R) and the method of Benjamini and Hochberg [41]. Significance was assumed for adjusted *P*-values equal to or below 0.1, if not stated otherwise. Statistical significance for bile acids and fatty acids was established using a Wilcoxon rank sum test. For determination of co-abundance groups, we first calculated the pairwise correlation matrix of microbial genera present in 37% of individuals using available python scripts for the programme SparCC [42]. The heatplot was then generated using the function heatplot with Ward linkage clustering, Pearson correlation distance and no scaling. PCoAs (Principal co-ordinates analysis) were determined using the Spearman-rank distance of faecal bile acid levels or faecal fatty acids, respectively.

## Results

### Clinical response to FMT

Ten patients underwent FMT in this study. Eigth were female, 2 were male, with a mean age of 54 years, and ages ranging from 22 to 90 years. The patients were given a stool transplant via gastroscopy to the duodenum as well as via colonoscopy to the distal ileum and right colon, as there was uncertainity with regard to the most effective route of delivery. The choice of bi-directional esophago-gastro-duodenoscopy and full colonoscopy was empirical to maximise the chance of success. Nine patients underwent antibiotic treatment prior to *C. difficile* associated diarrhoea, the remaining patient having pre-existing ulcerative colitis (Table 1). In terms of treatment regimens, 9 had undergone 3 or more antibiotic courses and 6 had undergone 4 or more. These courses included various regimens of the standard treatments, metronidazole, vancomycin and fidaxomicin. All patients had failed multiple courses of therapy for *C. difficile* with metronidazole, vancomycin and fidaxomicin prior to FMT. There was no antibiotic treatment given for 48 h before transplant. Nine out of ten patients improved clinically and remained *C. difficile* toxin negative for between 6 months to 2 years after a single FMT (Table 1). The patient who relapsed after FMT with material from a donor they selected had previously required colonic resection with ileorectal anastomosis for toxic megacolon due to *C. difficile*. This patient was found to have very high levels of glyco conjugated bile acid (~ 7500 μg/g) and taurine (~ 2500 μg/g) pre-FMT compared to most of the good responders of the transplant (who had very little or none of both glyco conjugated bile acid and taurine) (Additional file 1: Figure S4). Post FMT, the levels of both glyco-cojugated bile acids and taurine were reduced to < 500 μg/g for patient 1 (the initially failed FMT patient) (Additional file 1: Figure S4). However, this patient also improved clinically after a second FMT with a different donor.

### FMT moves the microbiota of recipients towards that of the donor and improves bacterial diversity

**Fig. 1:**
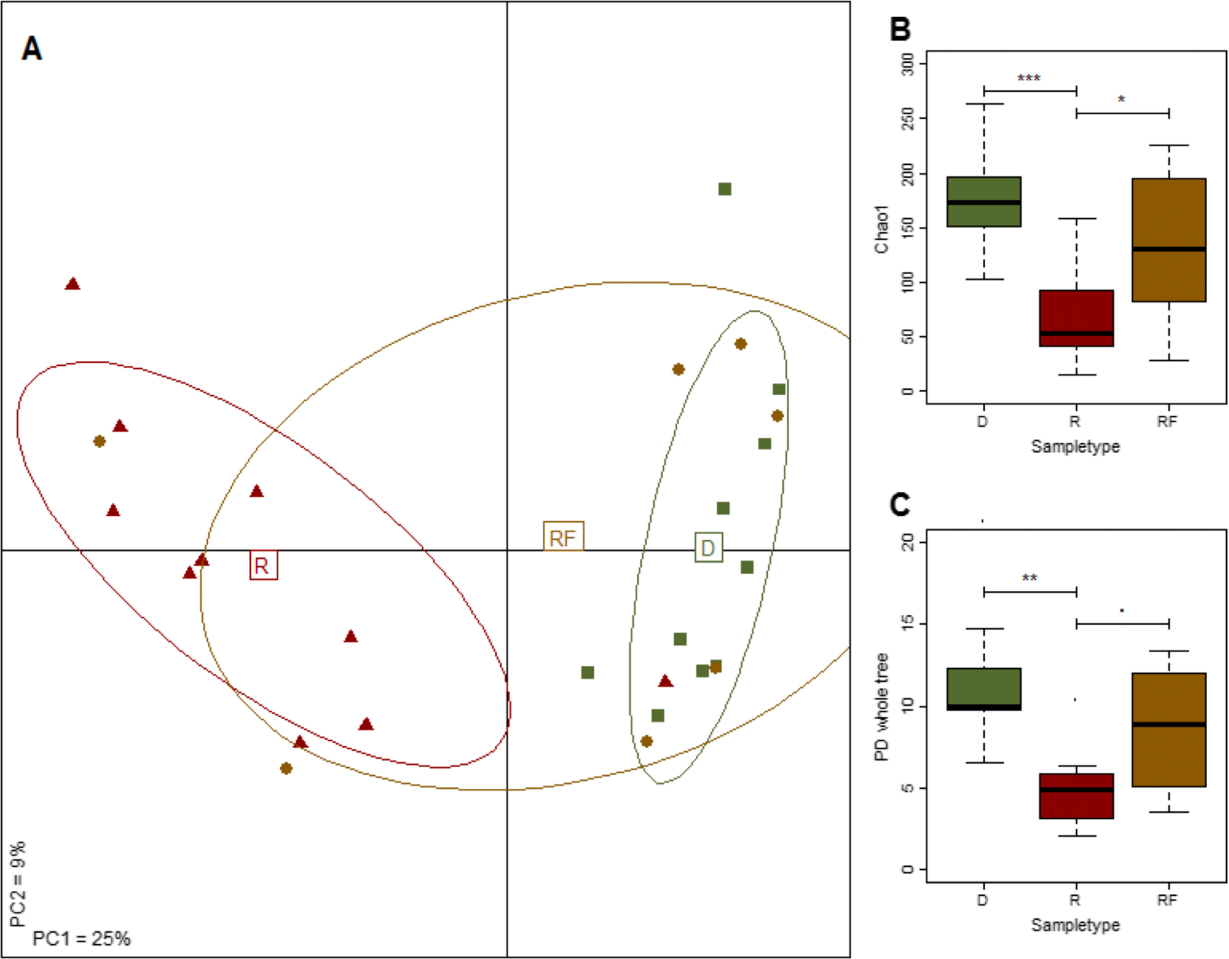
FMT restores the microbiota to a more donor-like profile. **a**: Overall the microbiota composition is more similar to the donor microbiota after FMT compared to before transplant. Shown is the PCoA (unweighted UniFrac distance) of the microbiota associated with CDI (“R”, *n* = 10), post-FMT (“RF”, *n* = 7) as well as FMT-donors (“D”, *n* = 10). The tenth sample can be found in the bottom-right part of the PCoA overlaping one of the RF samples. The microbiota prior to FMT is statistically significantly different, compared to the microbiota of donors (*P* < .001, F-statistic = 4.96, PERMANOVA), but assumes a more donor-like profile after FMT (*P* > .1, F-statistic = 1.22 for “RF” vs “D”). **b** and **c:** FMT leads to partial recovery of bacterial α-diversity. Shown, are box-and-whisker plots for the Chao1 index (**b**) and the phylogenetic diversity (PD whole tree) (**c**). Similar results were obtained for three other measures of bacterial α-diversity (Shannon- and Simpson-index, observed species). ‘***’ *P* < .001; ‘**’ *P* < .01; ‘*’ *P* < .05; ‘.’ *P* < .1

Microbiota profiles for 27 samples were obtained using 16S rRNA gene sequencing. Of these, 10 samples were obtained pre FMT (called “recipient” and designated “R” in Fig. 1), 7 were acquired between 1 and 4 months post FMT (“recipient-follow-up”/“RF”) and 10 samples were obtained from donors (“donor”/“D”). Only 7 follow-up samples were obtained as collection of the follow-up samples were time dependent and not all the patients could provide one. All patients who provided follow-up samples were in clinical remission when they provided the sample except for the first patient who failed the initial FMT and required a second with a unit from a different donor. Of the 10 donor samples, one was from the stool bank OpenBiome. The minimal sequencing depth per sample was 4211 reads. The overall microbiota of the patients with *C. difficile* infection was different from the donor microbiota (*p* < 0.001, PERMANOVA; Fig. 1 and Additional file 1: Figure S5). However, after FMT the composition of the microbiota was more similar to that of the donor (Fig. 1a and Additional file 1: Figure S5). Principal coordinates analysis of the unweighted UniFrac distances between the samples revealed that follow-up samples were more closely related to the donor than to the recipient (Fig. 1a and Additional file 1: Figure S5). This showed that FMT resulted in partial recovery of gut microbial community structure and the establishment of a more donor-like microbiota (Fig. 1a and Additional file 1: Figure S5). These compositional differences were also reflected in the bacterial diversity which we measured using several widely recognized measures of α-diversity: Chao1 index, Phylogenetic Diversity (Fig. 1b, c), and Simpson index, and observed species. For all indices, the alpha diversity was statistically significantly lower prior to FMT compared to donor samples (*P*-values from 2×10^− 4^ to 0.01, Student’s t-test) but recovered to levels observed in the donor samples post FMT (Fig. 1b). However, even though the diversity indices improved following FMT, the levels observed were still lower after the FMT-procedure compared to donor levels (73 - 88% of the donor-samples’ diversity index values, range of *P*-values from 0.05 to 0.19, depending on the index used) (Fig. 1b, c).

### Community structure and compositional changes in the microbiota at phylum and genus levels after FMT

Noteworthy differences at both bacterial phylum and genus levels were observed in recipient, follow-up and donor samples (Additional file 1: Figure S1 & Additional file 2: Table S6). The abundances of several individual genera or species clusters, including *Proteus*, *Sutterella*, *Fusobacterium*, Clostridium_XVIII, *Veilonella*, *Escherichia*/*Shigella*, *Klebsiella*, *Acidaminococcus* and *Streptococcus* (*P* < 1e-03, Wald test) were significantly higher in patients with *C. difficile* infection (Additional file 1: Figure S1) compared to donors. The levels of most of the aforementioned bacteria approached donor levels post-FMT indicating successful colonization and established communities from the donor microbiota. Only *Faecalibacterium* was found to be lower in the patients with *C. difficile* infection compared to the donors (*P* < 0.1).

**Fig. 2:**
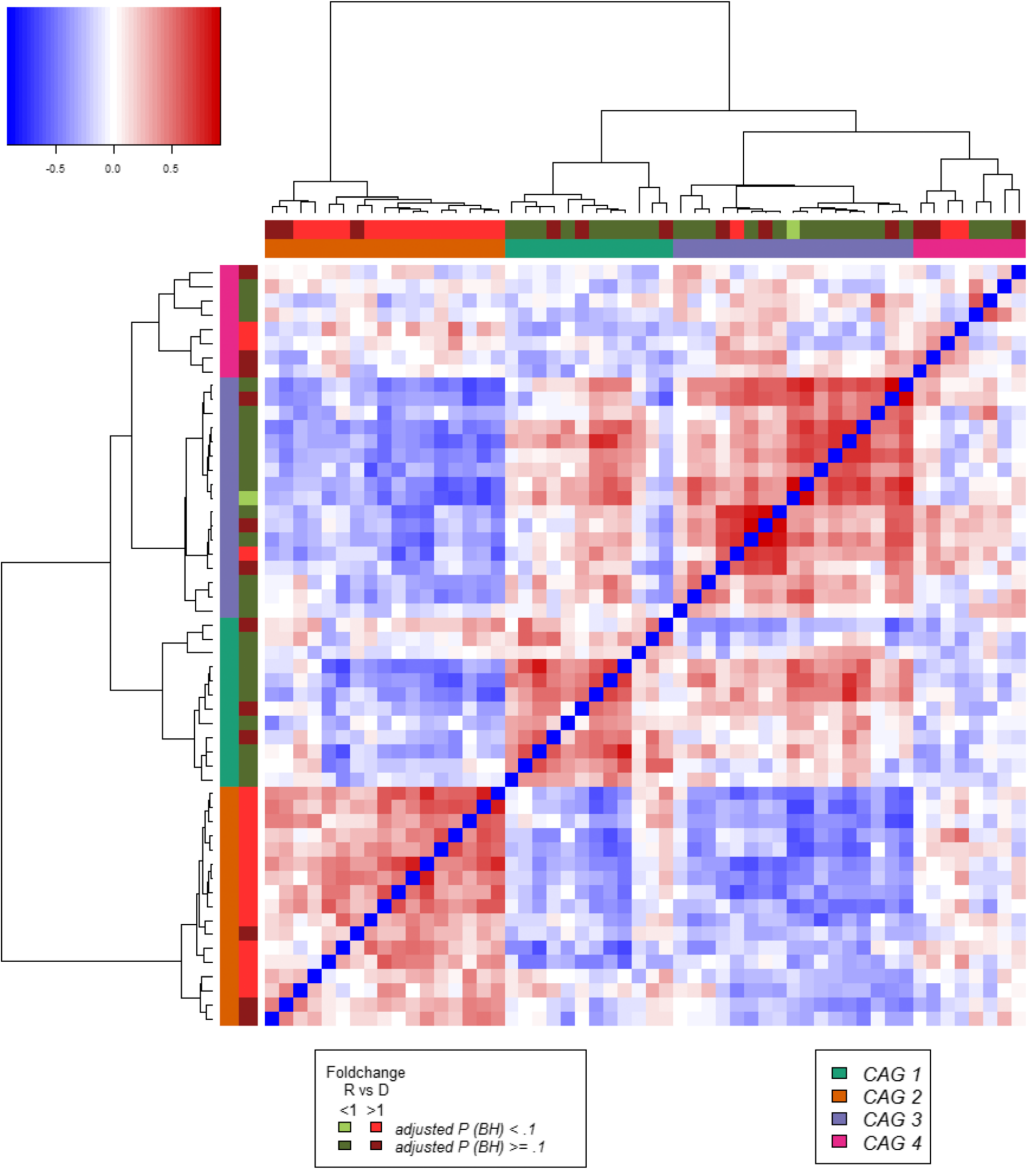
Hierarchical Ward-linkage clustering based on the correlation coefficients determined by SparCC. Co-abundance groups were defined on the basis of the clusters in the vertical tree. Colour coding of side bars: Co-abundance groups are coloured according to the legend at the bottom-right. Fold-change and statistical significance are coloured according to the legend at the bottom-left. Colour coding of the side bars according to the legends below. The composition of each Co-abundance Group is shown in Additional file 1: Figure S2

Further analysis of the microbiota was performed by determining Co-Abundance Groups (CAGs), i.e. clusters of co-occurring bacteria, an analytical approach employed productively in our previous microbiota analyses [31, 43, 44]. Four CAGs were identified based on the correlations between genera and determined by SparCC [42] (Fig. 2). Most bacterial genera with elevated abundance in recipients were found in one CAG (orange CAG 2 in Fig. 2, Additional file 1: Figures S2 & S3), reflecting similarities in terms of microbiota composition among patients. Contrastingly, bacteria with relatively high abundance in donors were grouped in CAG 1 and CAG 3 (turquoise and purple CAGs in Fig. 2, Additional file 1: Figures S2A & S3). The bacteria in these two CAGs are indicative of the presence of *Bacteroides* and *Ruminococcus*-like enterotypes [45], respectively, in the donor samples. A *Prevotella*-like enterotype was not detected in this cohort. We have noted that some RF samples still clustered with R samples (Fig. 1) indicating that microbiota community transplanted from the donor was not established properly in the recipient. Similarly, for some of the samples the bacterial α-diversity did not improve upon FMT treatment (Fig. 1b and c). We hypothesized that this could be explained by the microbiota profiles of either the donor or the recipient. To test this, we determined the change in terms of bacterial α-diversity induced by the FMT treatment and then correlated this change with the abundance of the four detected CAGs in the donor samples. The abundance of CAG 2 and CAG 3 in the donor samples was negatively (Spearman’s *ρ* = − 0.79, *p* = − 0.05) and positively (Spearman’s *ρ* = 0.82, *P* = 0.03) correlated, respectively, with the increase in bacterial α-diversity upon FMT treatment (Fig. 2 & Additional file 1: Figure S2). Additionally, the abundance of CAG 2 in donor samples was negatively associated with the abundance of CAG 3 in RF samples (Spearman’s *ρ* = − 0.82, *P* = 0.03) (Fig. 2 & Additional file 1: Figure S2). While this part of the analysis was not corrected for multiple testing due to the small sample size under consideration, this qualified preliminary observation may add to the rationale for choosing optimal donor samples for FMT.

### Faecal bile acid levels are significantly different in donors and recipients and are more donor-like in FMT recipients

**Fig. 3:**
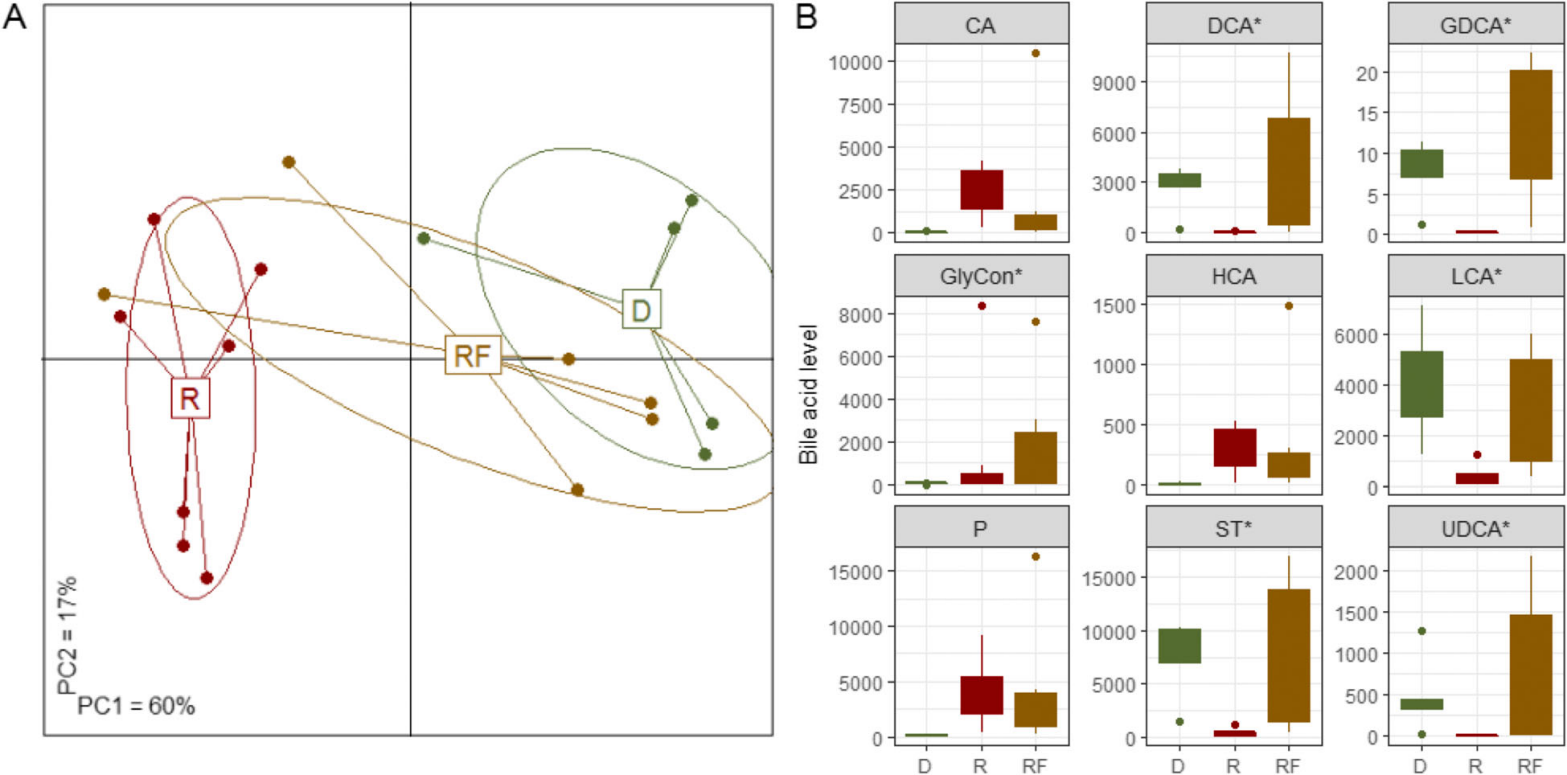
FMT restores faecal bile acids to more donor-like levels. **a**: Overall, the bile acid levels are more similar to those seen in the donor after FMT compared to before transplant. Shown is the PCoA (Spearman rank distance) of the bile acid levels associated with CDI (“R”, *n* = 7), post-FMT (“RF”, *n* = 6) as well as FMT-donors (“D”, *n* = 5). The bile acid profile before FMT is statistically significantly different compared to the microbiota of the donors (*P* < .01, PERMANOVA) but assumes a more donor-like profile after FMT (*P* > .1 for “RF” vs “D”). **b**: Many bile acids are restored to donor levels after FMT. Shown are boxplots for bile acids with differential abundance (*P* < .1) in patients with CDI (“R”) compared to the donors (“D”). Most of these faecal bile acids were not differentially abundant after the FMT procedure (“RF”) compared to the donors, indicated by asterix. D: donor, R: recipient, RF: recipient post-FMT, CA: DCA:, GDCA:, GlyCon: merged glyco-conjugated bile acids, HCA:, LCA:, P: merged primary bile acids, ST: merged secondary and tertiary bile acids, UDCA:, LCA:, DCA:, CA:, HCA:,GDCA

Faecal bile acids were quantified in samples from 5 donors, 7 patients and 6 recipients post-FMT. Analysis of the patients’ faecal bile acid levels revealed more donor-like profiles after FMT compared to before transplant (Fig. 3). The FMT effect in the majority of cases was to substantially increase and to restore the diversity of faecal bile acid levels (mean of 6651 μg/g in the recipient samples, 15,243 μg/g in the follow-up samples) (Fig. 3b). Here, similar to other studies, patients with *C. difficile* infection showed decreased levels of secondary bile acid, DCA, LCA and ursodeoxycholic acid. The levels of primary free bile acid were significantly elevated in individuals with *C. difficile* infection compared to donors (*P* < .05; Fig. 3b). This may account for the lower levels of overall bile acid in the faeces because CDCA is a potent ligand of farnesoid X receptor which signals through fibroblast growth factor 19 in the liver to lower bile acid synthesis [46–48]. Generally, tauro-conjugated bile acid levels were low (Fig. 3b) indicating that microbial bile acid deconjugation was unaffected in these individuals and, as with previously reported studies, 7α-dehydroxylation and epimerization by other microbial enzymes (from members of the Firmicutes containing the *bai* operon components) were depleted. Since the only experimentally confirmed 7α-dehydroxylation activity is found among *Clostridium* species, the use of *Clostridium* targeting antibiotics may be responsible for the loss of this activity. In combination with this, the use of elemental nutrition may also reduce *C. difficile* colonization resistance in these patients. *C. difficile* itself is equipped to deconjugate bile salts but does not have 7α-dehydroxylation ability. The levels of these secondary bile acid were restored post FMT and primary bile acid CDCA and CA decreased (Fig. 3b). Overall, glyco-conjugated bile acids levels were significantly higher in patients pre-FMT compared to donors (Fig. 3b). Taken together we can confirm that patients with *C. difficile* infection show a decreased secondary bile acid profile while upstream precursor molecules are elevated in these patients (Fig. 3b).

Interestingly, in assessing the bile acid signature in donor samples it is clear that different donors alter bile acid and salt moieties to different extents. Fresh material prepared under anaerobic condition appear capable of elevating secondary bile acid to a greater degree than frozen commercially available donor material (Additional file 1: Table S3). Furthermore, the level of conjugated bile acid in frozen material was 7-12 times lower than that of the fresh donor samples.

### Microbiota profiles strongly correlate with faecal bile acids

**Fig. 4:**
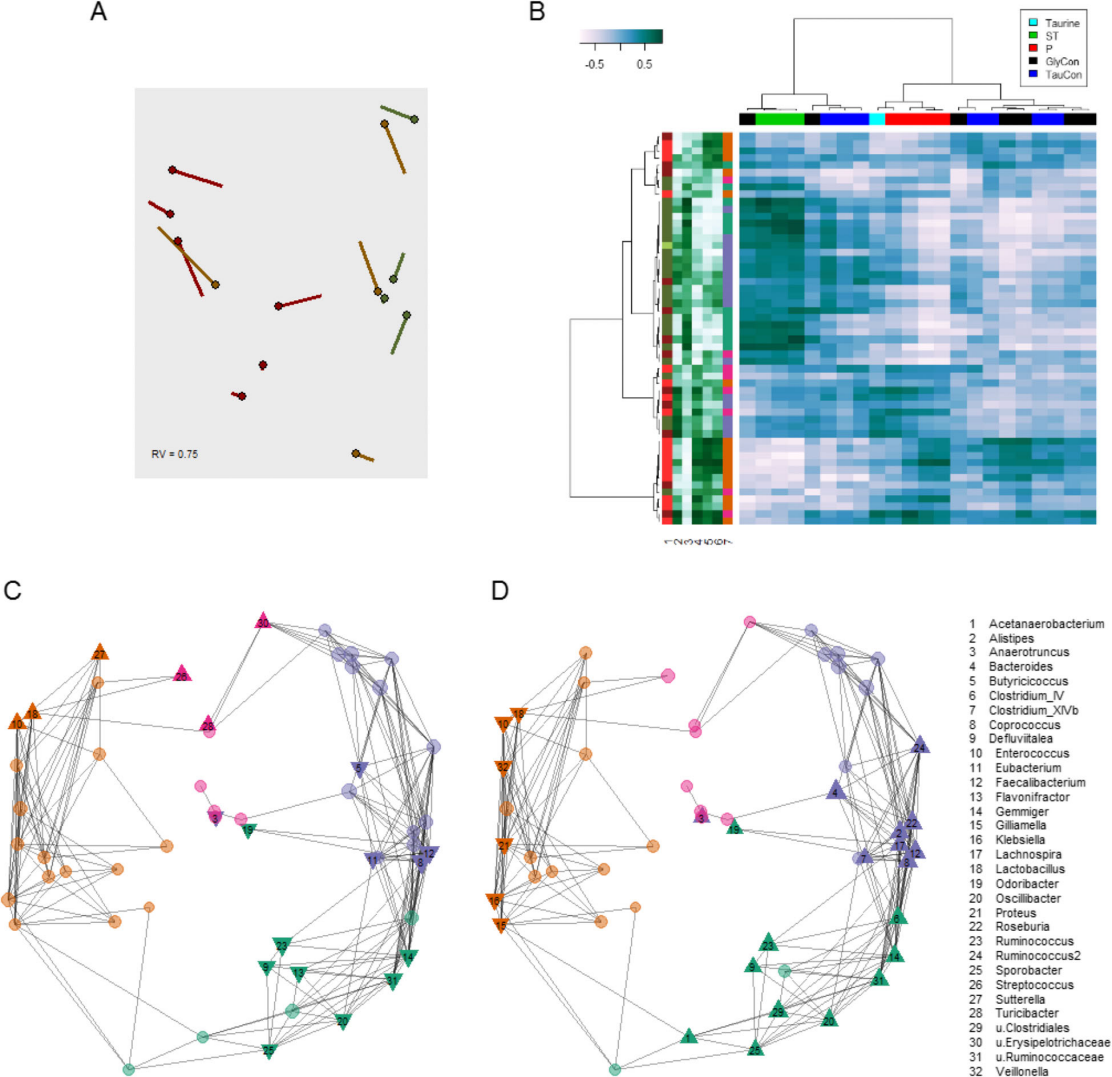
Bile acid and microbiota profiles are strongly correlated. **a** Co-inertia analysis of the microbiota and bile-acid based PCoAs from Additional file 1: Figures S1 and S4. Circles: location of sample is based on microbiota data; end of lines: location of sample is based on bile acid data; the length of each line is proportional to the difference between the bile acid and microbiota data; RV: measure of similarity on a scale from 0 to 1, the closer to 1, the greater the similarity between the two datasets. **b** Clusters of bacteria are strongly associated with bile acid levels, particularly primary (red column bar) and secondary and tertiary bile acids (green column bar). Shown is the heatmap of the Spearman correlations between the level of bile acids and bacterial genus log-ratio abundance (dark blue: positive correlation, white: negative correlation; scale in legend top-left). Column annotation as in legend top-right; row annotation (from left to right): *1* Fold change of bacterial abundance between patients pre-FMT and the donors (colours as in Fig. 2). *2-6* Spearman correlation coefficient between bacterial genera and combined bile acids. Dark green: positive correlation; white: negative correlation. From left to right: T, ST, P, GlyCon and TauCon. *7* CAG-membership for each bacterial genus (colours as in Fig. 2). **c** and **d** Network plots of bacterial genera. The sign of the Spearman correlation coefficient with primary faecal bile acids (**c**) and secondary and tertiary bile acids (**d**) is indicated for each genus. Upward-facing triangle: statistically significant positive correlation. Circle: not statistically significantly correlated. Downward facing triangle: statistically significant negative correlation. Colours of circles indicate CAG-membership of bacterial genera; colour code as in Fig. 2. The size of the circle or triangle is proportional to the mean relative abundance of the represented genus from all samples

Both microbiota and faecal bile acid data were available for 4 donors, 6 recipients pre-FMT and 4 recipients post-FMT. Co-inertia analysis of the microbiota and bile acid data based on the PCoAs shown in Figs. 1 and 3 was performed, and revealed a consistent relationship between the two datasets (Fig. 4a). Moreover, despite the small number of samples, both the overall profile of faecal bile acids as well as individual bile acids were statistically significantly correlated with the relative abundance of several bacterial genera (Fig. 4b-d).

We used network plots to examine the relationship between bacterial genera and bile acids, since gut bacteria are known to play a key role in hydrolysing primary to secondary bile acids, thereby conferring resistance to *C. difficile* infection [49]. The network plots (Fig. 4c and d) represent the relationship between bacterial genera and bile acid levels. Particularly interesting is that bacterial genera classified as *Anaerotruncus*, *Coprococcus*, *Defluviitalea*, *Faecalibacterium*, *Gemmiger*, *Odoribacter*, *Oscillibacter*, Ruminococcaceae, *Ruminococcus*, and *Sporobacter*, that were statistically significantly negatively associated with primary bile acid levels, were also statistically significantly positively related with secondary bile acid DCA and LCA levels (Fig. 4c and d). These genera are also known to differentially confer resistance to growth and germination of *C. difficile* [50–57]. Likewise, bacterial genera such as *Enterococcus* and *Lactobacillus* were found to be positively associated with primary bile acid levels and negatively related with secondary bile acid levels (Fig. 4c and d).

### Faecal fatty acid levels are significantly different in donors and recipients

**Fig. 5:**
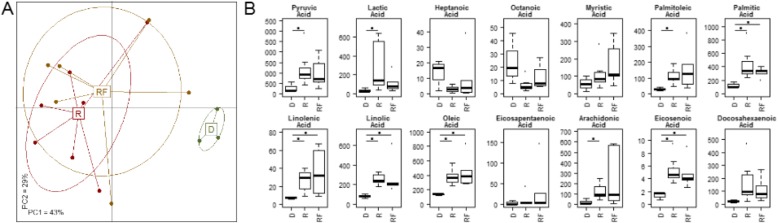
FMT does not restore faecal fatty acids to donor levels. **a** Donor-associated faecal fatty acids are distinct from recipients and follow-ups whereas faecal fatty acids do not change significantly after FMT. Shown is the principal coordinate analysis based on the faecal fatty acid levels (Spearman rank distance). **b** Boxplots showing levels of 14 faecal fatty acids. Statistical significance was calculated using a Wilcoxon rank sum test. *P*-values were adjusted for multiple testing using the method from Benjamini and Hochberg. Significance (*P* < .1) is indicated by circles. D: Donor (*n* = 3), R: Recipients (*n* = 7), RF: Follow-up (*n* = 4)

**Fig. 6:**
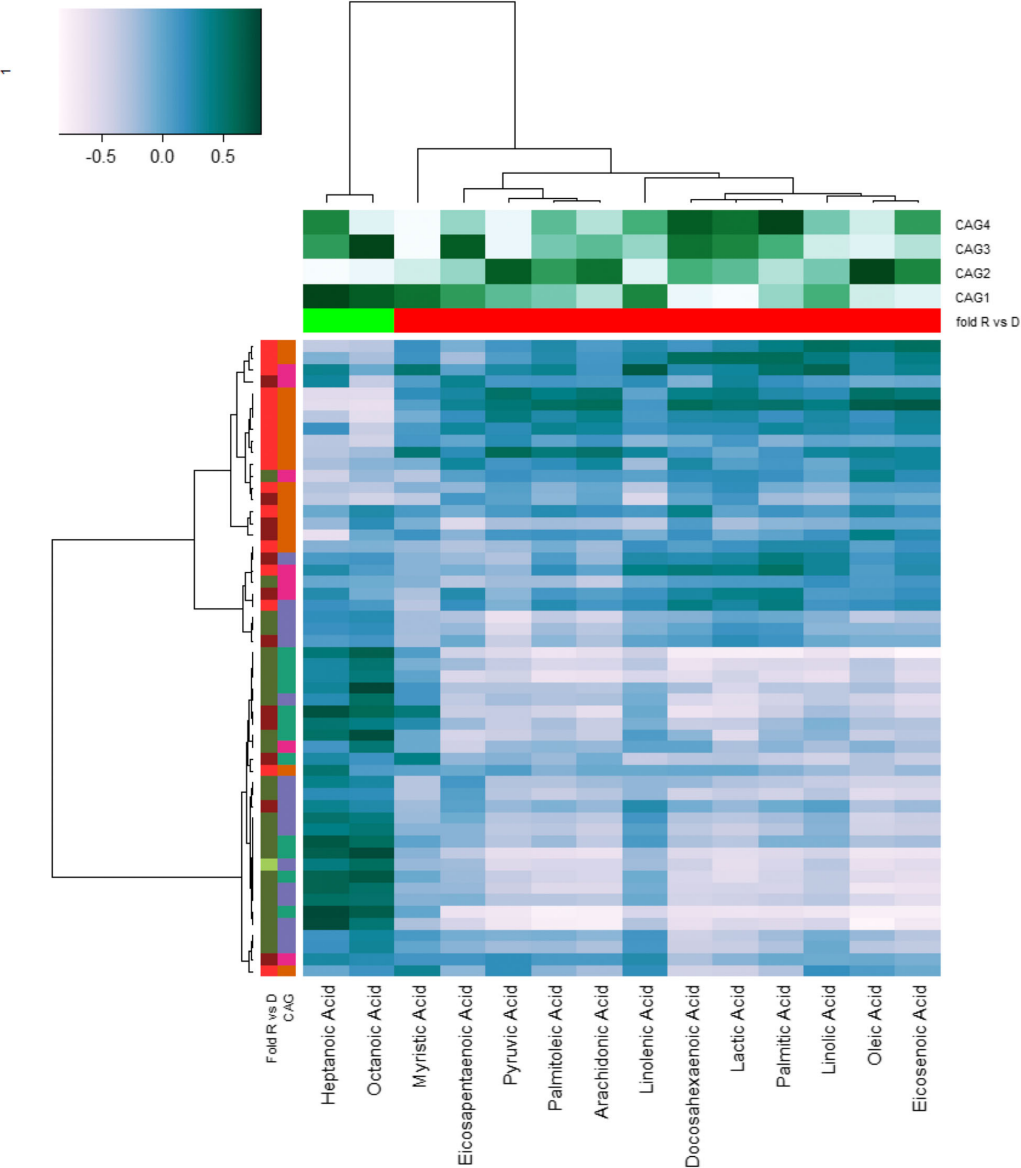
Faecal fatty acid levels are correlated with the abundance of several bacterial genera. Shown is the heat map of the Spearman correlations between faecal fatty acids (columns) and bacterial genus log-ratio abundance (rows). Column annotation: fold R vs D: fold-change of faecal fatty acids between recipients (“R”) and donors (“D”); red: > 1; green < 1. CAG1-4: Spearman correlation of fatty acid levels with log-ratio abundance of bacterial CAGs (Fig. 2). White: negative correlation, dark green: positive correlation. *Row annotation:* Left: Fold change of bacterial abundance between individuals pre-FMT (R) and donors (D). Right: CAG-membership for each bacterial genus. Colours for both as in Fig. 2

Inflammatory mediators are the link between innate and adaptive immunity. Inappropriate damage to host tissue through uncontrolled inflammation via pathogen toxins or endotoxins is proposed to release cellular fatty acids in CD infection [58]. Fatty acids, particularly PUFA metabolised to eicosanoids, are a key factor to mediate and regulate the inflammation response [59]. ω-3 PUFA and arachidonic acid is a key precursor of inflammatory cells and its levels dictate the duration and intensity of the response. On the other hand, ω-6 PUFA Docosahexaenoic acid (DHA) and Eicosapentaenoic acid (EPA) moderate the effect of arachidonic acid and its downstream products through anti-inflammatory resolvins, docosatrienes, neuroprotectins for DHA and E series resolvins via EPA. Since there is a proposed link between PUFAs and gut health [60, 61], we decided to profile a number of FAs. Individuals with *C. difficile* infection showed differences in the levels of 14 FAs examined relative to healthy donors, and PCoA analysis did not demonstrate overall conversion of FA profiles to the donor type in recipients (*p* > .1, PERMANOVA; Fig. 5a). Although reduced levels of short chain fatty acids, butyrate, propionate and acetate, have been previously reported by Seekatz et al., [62], we found elevated levels of fatty acids in patient groups relative to donors. This included SCFA, pyruvic acid and lactic acid, saturated fatty acids palmitic acid, and myristic acid – also an ω-6 FA, monounsaturated FAs, oleic acid and palmitoleic acid, ω-3 PUFAs α-linolenic acid and γ-linolenic acid (ALA+GLA) and EHA and DHA as well as, ω-6 Linoleic acid and arachidonic acid (Fig. 5a). There was some indication of a normalisation of individual inflammatory FA levels in recipients. SCFA, ω-3 & ω-6 PUFAs were altered in the direction of the donor following FMT (Fig. 6b). The relative ratios of detected anti-inflammatory ω-3 to pro-inflammatory ω-6 PUFAs in healthy donors was 1:1.3, for the *C. difficile* patient group this ratio was 1:1.8 and this ratio was altered post FMT to 1: 1.4. Taken together these data indicate that patients with *C. difficile* infection show higher levels of FA that may be indicative of inflammatory cell recruitment as well as liberation of cell membrane arachidonic acid or of increased dietary fermentation by microbes. Although FA levels remain high the relative dilution effect of ω-3 PUFA is evident post FMT and may be taken as an indicator or biomarker of reduced inflammation post FMT.

### Archaeal abundance revealed by metagenomic sequencing

Detection of archaeal taxa by bacterial 16S rRNA gene primers is unreliable [63]. We performed metagenomic shotgun sequencing for 4 follow-up samples post FMT and 4 donor samples (1.97 × 10^7^ mean read count after quality filtering, 2.73 × 10^5^ standard deviation). Similar sample stratification as for the microbiota profiles was obtained using functional profiles (Additional file 1: Figure S6) and we found that genes associated with archaeal metabolism were enriched in the follow-up samples, post FMT (Additional file 1: Figure S6). More detailed analysis showed that the contributing microorganisms were either *Methanobrevibacter smithii* or *Methanosphaera stadtmanae* (Additional file 3: Table S7). Curiously, when genes for archaeal metabolism were detected in a follow-up sample, archaea were of lower abundance in the respective donor sample or not found at all (Additional file 1: Figure S6). Moreover, for one paired set, archaeal metabolism genes were found in the donor sample but not in the follow-up sample (Additional file 1: Figure S6).

## Discussion

In this study, we established faecal microbiota changes that correlated with bile and faecal fatty acid profiles of 10 patients with relapsing *C. difficile* infection after they successfully received therapeutic FMT. Patients with *C. difficile* overgrowth had a distinct low diversity microbiome prior to FMT, but acquired a more diverse microbiota resembling the donors after transplantation. Interestingly, while mean *α*-diversity was significantly higher in FMT recipients, values were not completely restored to donor levels, and the degree of restoration correlated with the representation of certain co-abundance groups in the donor sample. This may allow FMT donor stratification or selection for efficacy in future. Additionally, in support of this we describe the case of patient FMT01 who experienced a failed FMT with their own selected donor, and a microbiota comprising of CAG 2 (consisting mainly the phylum Proteobacteria), post-transplant, compared to a good responder such as FMT07, where the microbiota was more diverse, consisting of CAGs 1, 2 and 3 (Additional file 1: Figures S2 & S3).

Age is known to be one of the predisposing factors for the development of *C. difficile* infection, because older people have a microbiota that is apparently permissive. Studies carried out on elderly cohorts have reported an increase in *Bacteroidetes* and *Proteobacteria* and a decrease in some *Firmicutes* as well as *Bifidobacteria* [43]. Previous studies have reported that the presence in FMT patients of the following taxa, Bacteroides *spp*., *Alistipes*, *Ruminococcaceae*, *Clostridium cluster IV*, *Clostridium cluster XIVa*, *Lachnospiraceae, Peptostreptococcaceae* and *Verrucomicrobiaceae* are protective against *C. difficile* colonization, whereas *Lactobacillus* spp., *Streptococcaceae*, *Enterobacteriaceae*, *Veillonella*, *Enterococcus* spp., *Salmonella* spp. and *Sutterella* spp., were associated with susceptibility [26, 53, 64–68]. We observed increased abundances of *Clostridia* and *Bacilli* as well as members of the phylum *Bacteroidetes* in samples from healthy donors (Additional file 1: Figure S1. CAGs 1 & 3) compared to patients with *C. difficile* infection [69]. The largest groups within these two phyla were *Clostridia* and *Bacteroidia*. Depletion of key bacterial species within these two phyla may reduce protection against *C. difficile* associated diarrhoea.

Higher faecal levels of primary bile acids were measured in patients with recurrent *C. difficile* infection prior to FMT in comparison to the donors (Fig. 3, Additional file 1: Figure S4) and these levels decreased following FMT. This is consistent with findings of a previous study [64]. After FMT, we noted that the levels of secondary bile acids were restored towards levels seen in the donor. We also observed that the levels of secondary bile acids detected in the faecal sample of a good responder to FMT post-transplant were more than twice the levels found in the patient with the failed procedure (~ 13,000 μg/g for the former and ~ 5000 μg/g for the latter) Additional file 1: Figure S4. Secondary bile acids are exclusively produced by bacterial metabolism in the large intestine. Primary bile acids such as CA and taurocholate promote germination of *C. difficile* spores, whereas secondary bile acids LCA generally inhibit germination and growth [70–72]. Alteration in the normal microbiota of the gut by antibiotic treatment, in particular the elimination of species capable of 7α-dehydroxylation of primary bile salts, causes an increase in the concentration of CA primary bile salts and a decrease in the concentration of secondary bile salts (DCA) [73]. This in turn affects *C. difficile* germination and growth. Theriot et al. also reported that changes in the microbial community structure of mice treated with antibiotics correlated with shifts in secondary bile acids [51]. Elevated free and glyco-conjugates of CDCA have the effect of inhibiting germination of CD spores, an effect that can also be blocked by the secondary bile acid ursodeoxycholic acid, while vegetative growth is enhanced by deoxycholic acid [70, 74, 75]. Interestingly CA and CDCA can alter Na+/K+ channel activation to prevent removal of water in the gastrointestinal tract and therefore induce bile acid diarrhoea, contributing to the disease [76].

The microbial populations present in the lower GI tract dictate the bile acid signatures that can either promote or prevent *C. difficile* outgrowth and colonization, effects that are resolved through FMT via both bile acid alteration to prevent outgrowth, and by colonization resistance restoration through the microbiota. Resolution or recovery from *C. difficile* infection may also be associated with the successful transfer of 7α-dehydroxylation bile acid inducible *C. difficile* (*baiCD*) genes. Solbach et al. reported reduced prevalence of the *baiCD* gene cluster in *C. difficile* patients and found successful FMT therapy in 1 out of 2 patients with recurrent *C. difficile* infection was associated with acquisition of *baiCD* genes [77].

We identified some normalisation of fatty acid profiles after FMT which correlated positively with Firmicutes abundance (the main contributor to taxa comprizing CAGs 1 and 3 in Fig. 6). This normalization comprised of elevation in the levels of some monounsaturated fatty acids and reduction in long chain polyunsaturated fatty acid levels. It may be that some of these long chain fatty acids are being metabolised by *Firmicutes* in syntrophy with gut microbes to generate energy. The mechanistic role of this in symptomatic recovery remains uncertain. We also acknowledge that the numbers in our study are small and the effect of fatty acid is very modest.

The finding of genes for archaeal metabolism in a follow-up sample, and their lower abundance or absence in the respective donor sample, as well as the presence of genes in the donor but not in the follow up sample, is intriguing (Additional file 3: Table S7). Methanogens are sensitive to drugs commonly used to treat *C. difficile* such as metronidazole [78] and it is likely they are routinely eliminated in *C. difficile* patients as a by-product of standard therapy prior to transplant. They are not present in all people and are also adversely affected by statins. Most bacteria are not affected by statins which can however specifically interfere with cell membrane synthesis in archaea [79]. Donors and recipients were not screened for non-antibacterial medication. One explanation for the lack of methanogen congruence between donor and recipients could be that both groups may contain individuals with low level methanogen colonization due to suppression by statin therapy.

In summary, FMT restores the microbiota of patients with *C. difficile* infection to a state closer to that of the donor in respect of alpha diversity and co-abundance group microbiota structure, while it effectively treats the illness. A major metabolic signature of this transformation is a reduction in levels of primary bile acids and an increase in levels of secondary bile acids, both of which are likely to impact adversely on *C. difficile* growth and germination. We have confirmed previous reports that specific bile acid profiles are associated with *C. difficile* associated diarrhoea. Related to this, we found that the combination of key genera such as *Ruminococcus* and *Bacteroides* appears to be inherently linked to the metabolism of secondary bile acids and would therefore be protective against *C. difficile* colonisation. A lesser effect was noted for fatty acids. Lastly, FMT had a limited effect on archaeal ecology in the recipient samples although we could not verify this from the 16S data. While FMT as a treatment of *C. difficile* infection is usually successful and was so in the current study, a short-term effect of archaea is not apparent in this context, but long-term effects may be possible.

## Conclusions

Patients whose *C. difficile* infection was successfully treated by faecal transplantation experienced a microbiota shift towards that of the donor with a metabolic signature reflecting normalisation of bile acid metabolism that may be the mechanism of transplantation efficacy. An effect on the ratios of inflammatory and non-inflammatory FAs was also noted. Donor microbiota taxa associated with restoration of bile acid metabolism and microbial diversity were variably present in donors, suggesting rational donor selection may be possible.

## Additional files

Additional file 1: **Table S1.** Primer sequences used in this study. **Table S2.** Bile acids identified by UPLC-MS analysis. **Table S3.** Bile acid profile of fresh donor samples vs frozen donor. **Table S4.** Fatty acids identified by UPLC-MS analysis. **Table S5.** R Libraries and the Versions. **Figure S1.** The relative abundance of many bacterial genera is restored to donor levels after FMT. **Figure S2**. Composition of the 4 Co-abundance groups in Fig. 2, A: Classification at genus level. B: Classification at phylum level. **Figure S3.** The relative abundance of Co-abundance Groups across all samples. **Figure S4.** Faecal bile acid levels in all the samples. **Figure S5.** PCoA of the microbiota based on the weighted UniFrac distance. **Figure S6.** Heatmap based on metagenomic shotgun sequencing. (DOCX 428 kb)

Additional file 2: **Table S6.** Taxonomic count of shotgun data for this study. (XLSX 19 kb)

Additional file 3: **Table S7.** Genes associated with archaeal metabolism obtained from shotgun metagenomic sequencing (XLS 24 kb)

## Abbreviations

*C. difficile*: Clostridioides difficile
CA: Cholic acid
CAG: Co-Abundance group
CDCA: Chenodeoxycholic acid
DCA: Deoxycholic acid
DHA: Docosahexaenoic acid
DNA: Deoxyribonucleic acid
EPA: Eicosapentaenoic acid
FA: Fatty acid
FMT: Faecal microbiota transplantation
LCA: Lithocholic acid
PCoA: Principle co-ordinate analysis
PCR: Polymerase chain reaction
PUFA: Polyunsaturated fatty acid
rRNA: Ribosomal ribonucleic acid
SCFA: Short chain fatty acid
