# Supplementary material for: Changes in microbiota composition, bile and fatty acid metabolism, in successful faecal microbiota transplantation for Clostridioides difficile infection
Source: BMC Gastroenterol. 2018 Aug 28;18:131. doi: 10.1186/s12876-018-0860-5 (PMC6114236; doi:10.1186/s12876-018-0860-5)
Supplement: Supplementary file 1 — Table S1. Primer sequences used in this study. Table S2. Bile acids identified by UPLC-MS analysis. Table S3. Bile acid profile of fresh donor samples vs frozen donor. Table S4. Fatty acids identified by UPLC-MS analysis. Table S5. R Libraries and the Versions. Figure S1. The relative abundance of many bacterial genera is restored to donor levels after FMT. Figure S2. Composition of the 4 Co-abundance groups in Fig. 2, A: Classification at genus level. B: Classification at phylum level. Figure S3. The relative abundance of Co-abundance Groups across all samples. Figure S4. Faecal bile acid levels in all the samples. Figure S5. PCoA of the microbiota based on the weighted UniFrac distance. Figure S6. Heatmap based on metagenomic shotgun sequencing. (DOCX 428 kb) [file 12876_2018_860_MOESM1_ESM.docx]

**Additional file 1**

**Table S1 16S** Primer sequences used in this study

| Primer Name | Sequence (5'-3') |
| --- | --- |
| V3 Forward Primer | TCGTCGGCAGCGTCAGATGTG-TATAAGAGACAGCCTACGGG-NGGCWGCAG |
| V4 Reverse Primer | GTCTCGTGGGCTCGGAGATGTG-TATAAGAGACAGGACTACHVGGG-TATCTAATCC |
| \| N701(FP) \| S520(RP) \| \| --- \| --- \| | TAAGGCGAAAGGCTAT |
| \| N701(FP) \| S521(RP) \| \| --- \| --- \| | TAAGGCGAGAGCCTTA |
| \| N701(FP) \| S522(RP) \| \| --- \| --- \| | TAAGGCGATTATGCGA |
| \| N702(FP) \| S513(RP) \| \| --- \| --- \| | CGTACTAGTCGACTAG |
| \| N702(FP) \| S515(RP) \| \| --- \| --- \| | CGTACTAGTTCTAGCT |
| \| N702(FP) \| S520(RP) \| \| --- \| --- \| | GCTACTAGAAGGCTAT |
| \| N702(FP) \| S521(RP) \| \| --- \| --- \| | CGTACTAGGAGCCTTA |
| \| N702(FP) \| S522(RP) \| \| --- \| --- \| | CGTACTAGTTATGCGA |
| \| N702(FP) \| S516(RP) \| \| --- \| --- \| | CGTACTAGCCTAGAGT |
| \| N702(FP) \| S517(RP) \| \| --- \| --- \| | CGTACTAGGCGTAAGA |
| \| N702(FP) \| S518(RP) \| \| --- \| --- \| | CGTACTAGCTATTAAG |
| \| N703(FP) \| S518(RP) \| \| --- \| --- \| | AGGCAGAACTATTAAG |
| \| N714(FP) \| S507(RP) \| \| --- \| --- \| | GCTCATGAAAGGAGTA |
| \| N702(FP) \| S507(RP) \| \| --- \| --- \| | CGTACTAGAAGGAGTA |
| \| N702(FP) \| S508(RP) \| \| --- \| --- \| | CGTACTAGCTAAGCCT |
| \| N702(FP) \| S510(RP) \| \| --- \| --- \| | CGTACTAGCGTCTAAT |
| \| N705(FP) \| S503(RP) \| \| --- \| --- \| | GGACTCCTTATCCTCT |
| \| N705(FP) \| S505(RP) \| \| --- \| --- \| | GGACTCCTGTAAGGAG |
| \| N705(FP) \| S506(RP) \| \| --- \| --- \| | GGACTCCTACTGCATA |
| \| N705(FP) \| S507(RP) \| \| --- \| --- \| | GGACTCCTAAGGAGTA |
| \| N705(FP) \| S508(RP) \| \| --- \| --- \| | GGACTCCTCTAAGCCT |
| \| N705(FP) \| S510(RP) \| \| --- \| --- \| | GGACTCCTCGTCTAAT |

**Table S2** Bile acids identified by UPLC-MS analysis


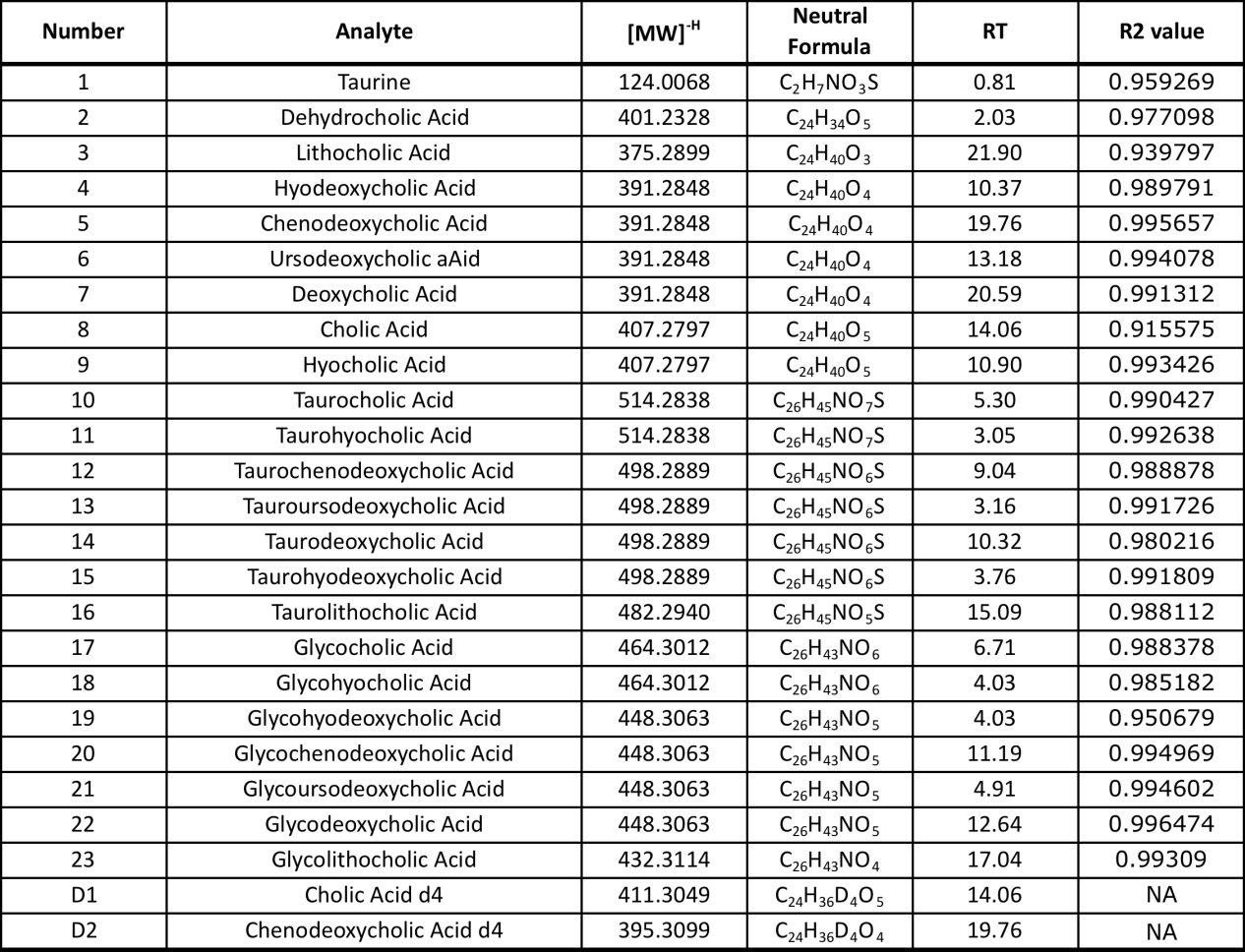


**Table S3** Bile acid profile of fresh donor samples vs frozen donor.

| Bile Acid | FTD 007 | OPEN BIOME (FTD 010) | FTD 006 |
| --- | --- | --- | --- |
| Taurine | 150.07 | 29.21 | 152.40 |
| LCA | 7126.13 | 1230.45 | 5268.66 |
| DCA | 2773.91 | 232.36 | 3550.45 |
| UDCA | 340.74 | 35.63 | 1282.73 |
| CA | 36.98 | 9.97 | 42.96 |
| CDCA | 82.58 | 8.32 | 143.21 |
| HDCA | 5.91 | 3.55 | 9.47 |
| HCA | 5.89 | 1.82 | 4.82 |
| GCDCA | 14.78 | 2.11 | 15.49 |
| GCA | 13.07 | 1.63 | 8.22 |
| GHCA | 11.44 | 1.51 | 8.67 |
| GHDCA | 2.73 | 0.66 | 4.64 |
| GUDCA | 0.38 | 0.41 | 2.23 |
| GDCA | 11.29 | 1.34 | 6.98 |
| GLCA | 9.76 | 0.42 | 9.84 |
| TCDCA | 9.48 | 0.62 | 7.03 |
| TUDCA | 8.84 | 0.19 | 0.44 |
| TDCA | 2.80 | 0.79 | 6.49 |
| THDCA | 0.74 | 0.15 | 3.16 |
| TCA | 8.08 | 0.36 | 4.45 |
| TLCA | 1.55 | 2.25 | 8.18 |
| THCA | 0.46 | 0.21 | 3.54 |

**Table S4** Fatty acids identified by UPLC-MS analysis

| **Analyte** | **[MW]^-H^** | **Neutral Formula** | **RT** | **R value** |
| --- | --- | --- | --- | --- |
| Pyruvic acid | 87.0082 | C_3_H_4_O_3_ | 0.81 | 0.8712 |
| Lactic acid | 89.0239 | C_3_H_6_O_3_ | 0.8 | 0.827 |
| Heptanoic acid | 129.0916 | C_7_H_14_O_2_ | 2.95 | 0.9018 |
| Octanoic acid | 143.1072 | C_8_H_16_O_2_ | 4.93 | 0.93 |
| Myristic acid | 227.2011 | C_14_H_28_O_2_ | 22.15 | 0.8059 |
| Palmitoleic acid | 253.2168 | C_16_H_30_O_2_ | 22.25 | 0.9577 |
| Palmitic acid | 255.2324 | C_16_H_32_O_2_ | 22.55 | 0.9815 |
| Linolenic acid | 277.2168 | C_18_H_30_O_2_ | 22.15 | 0.9115 |
| Linoleic acid | 279.2324 | C_18_H_32_O_2_ | 22.34 | 0.8765 |
| Oleic acid | 281.2481 | C_18_H_34_O_2_ | 22.59 | 0.9123 |
| cis-5,8,11,14,17-Eicosapentaenoic acid | 301.2168 | C_20_H_30_O_2_ | 22.11 | 0.9484 |
| Arachidonic acid | 303.2324 | C_20_H_32_O_2_ | 22.3 | 0.9669 |
| Cis-11-Eicosenoic acid | 309.2794 | C_20_H_38_O_2_ | 22.97 | 0.8664 |
| Cis-4,7,10,13,16,19-Docosahexaenoic acid | 327.2324 | C_22_H_32_O_2_ | 22.25 | 0.8965 |

**Table S5 R Libraries and the Versions**

# Version and system information

## R version 3.3.2 (2016-10-31)
## Platform: x86_64-w64-mingw32/x64 (64-bit)
## Running under: Windows 7 x64 (build 7601) Service Pack 1
##
## locale:
## [1] LC_COLLATE=English_Ireland.1252 LC_CTYPE=English_Ireland.1252
## [3] LC_MONETARY=English_Ireland.1252 LC_NUMERIC=C
## [5] LC_TIME=English_Ireland.1252
##
## attached base packages:
## [1] stats graphics grDevices utils datasets methods base
##
## loaded via a namespace (and not attached):
## [1] SummarizedExperiment_1.2.3 genefilter_1.54.2
## [3] locfit_1.5-9.1 splines_3.3.2
## [5] lattice_0.20-34 colorspace_1.3-2
## [7] htmltools_0.3.6 stats4_3.3.2
## [9] yaml_2.1.14 base64enc_0.1-3
## [11] survival_2.39-5 XML_3.98-1.6
## [13] foreign_0.8-68 DBI_0.6-1
## [15] BiocParallel_1.6.6 BiocGenerics_0.18.0
## [17] RColorBrewer_1.1-2 plyr_1.8.4
## [19] stringr_1.2.0 zlibbioc_1.18.0
## [21] munsell_0.4.3 gtable_0.2.0
## [23] DESeq2_1.12.4 htmlwidgets_0.8
## [25] evaluate_0.10 memoise_1.1.0
## [27] latticeExtra_0.6-28 Biobase_2.32.0
## [29] knitr_1.15.1 geneplotter_1.50.0
## [31] IRanges_2.6.1 GenomeInfoDb_1.8.7
## [33] parallel_3.3.2 AnnotationDbi_1.34.4
## [35] htmlTable_1.9 Rcpp_0.12.10
## [37] acepack_1.4.1 xtable_1.8-2
## [39] scales_0.4.1 backports_1.0.5
## [41] checkmate_1.8.2 S4Vectors_0.10.3
## [43] Hmisc_4.0-3 annotate_1.50.1
## [45] XVector_0.12.1 gridExtra_2.2.1
## [47] ggplot2_2.2.1 digest_0.6.12
## [49] stringi_1.1.5 GenomicRanges_1.24.3
## [51] grid_3.3.2 rprojroot_1.2
## [53] tools_3.3.2 bitops_1.0-6
## [55] magrittr_1.5 lazyeval_0.2.0
## [57] RCurl_1.95-4.8 tibble_1.3.0
## [59] RSQLite_1.1-2 Formula_1.2-1
## [61] cluster_2.0.6 Matrix_1.2-8
## [63] data.table_1.10.4 rmarkdown_1.5
## [65] rpart_4.1-11 nnet_7.3-12

## sysname release
## "Windows" "7 x64"
## version nodename
## "build 7601, Service Pack 1" "POTOOLE-PC"
## machine login
## "x86-64" "Burk"
## user effective_user
## "Burk" "Burk"


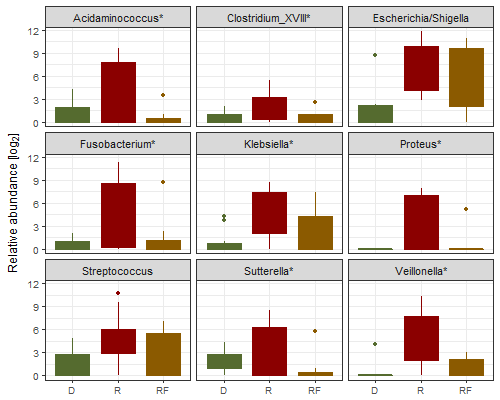


**Figure S1**: The relative abundance of many bacterial genera is restored to donor levels after FMT. Shown are boxplots for genera with statistically significantly higher abundance (*P* < .001, Wald-test) in patients with CDI (recipients) compared to donors. Most of these genera were not differentially abundant after the FMT procedure (follow-up) compared to donors, indicated by asterix. D: Donor, R: Recipient, RF: Recipient post-FMT.

A


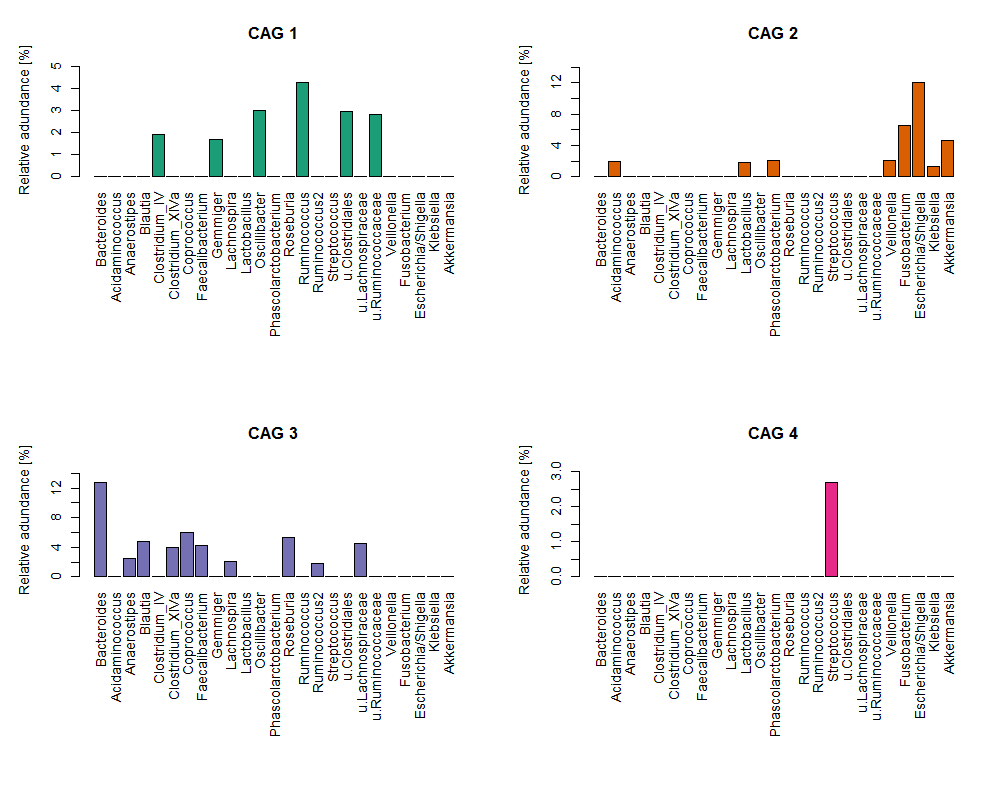


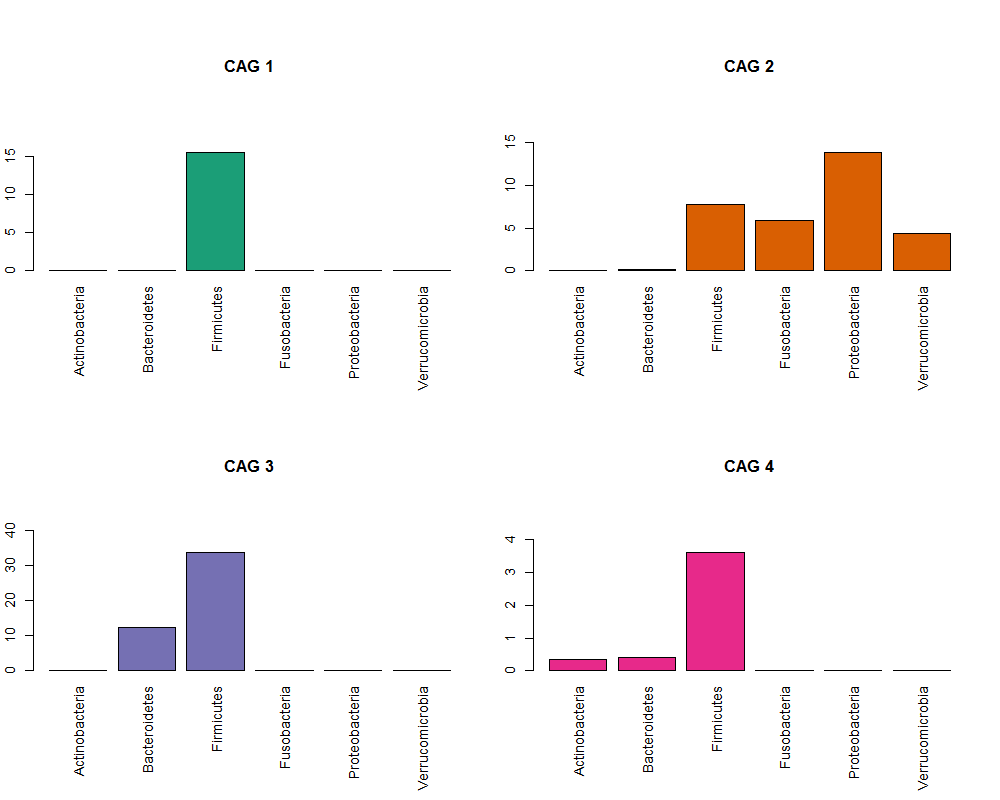


B

**Figure S2:** Composition of the 4 Co-abundance groups in Figure 2. **A**: Classification at genus level. **B:** Classification at phylum level.


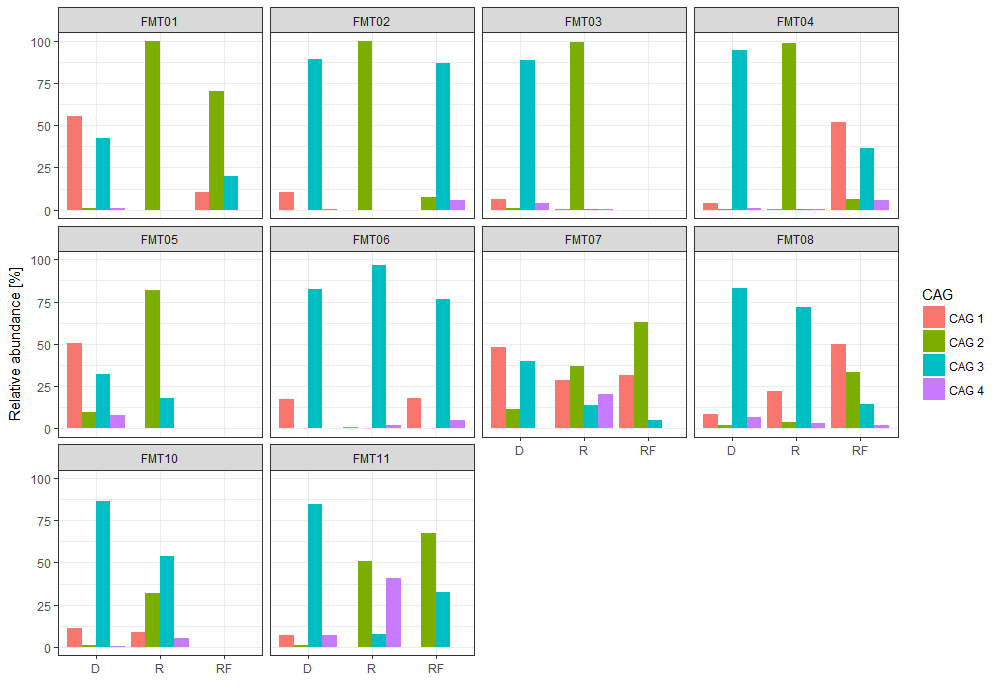


**Figure S3:** The relative abundance of Co-abundance Groups across all samples. Colour coding of CAGs (Figure 2) according to legend to the right. D = donor, R = recipient, RF = follow-up.


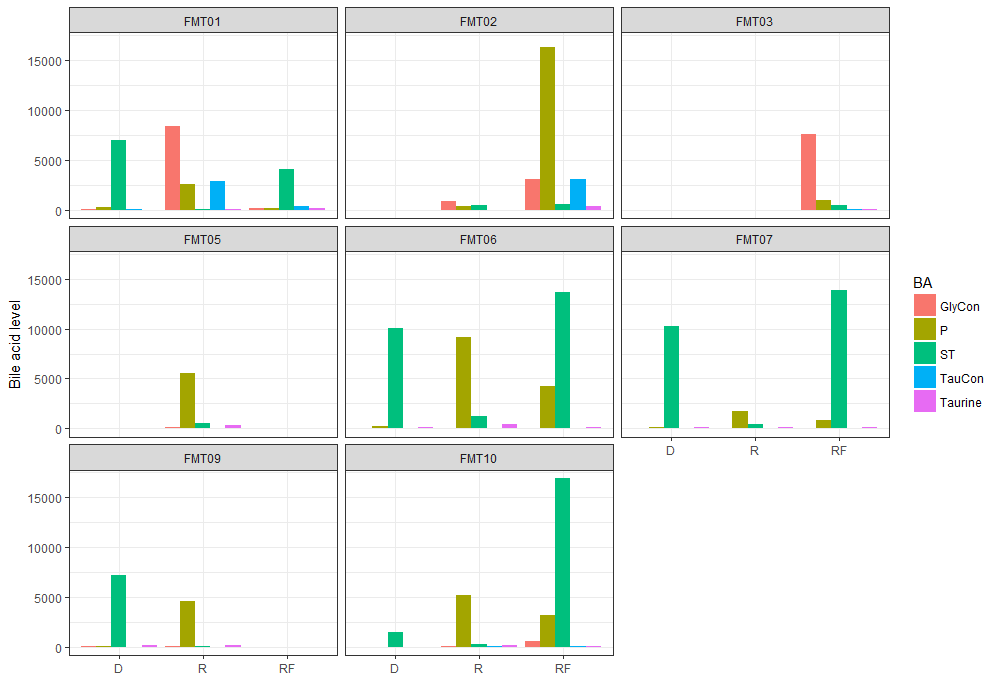


**Figure S4:** Faecal bile acid levels in all the samples. The levels obtained for single bile acids (see Supplementary Table 2) were combined to glyco-conjugated bile acids (GlyCon), primary bile acids (P), secondary and tertiary bile acids (ST), tauro conjugated bile acids (TauCon) and taurine bile acids. Colour coding is according to the legend at the right. D = donor, R = recipient, RF = follow-up.


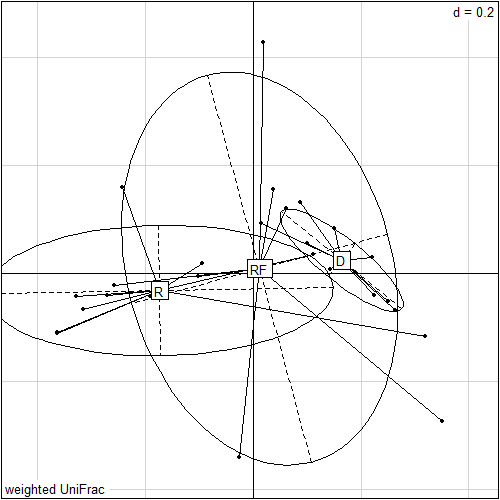


**Figure S5:** PCoA of the microbiota based on the weighted UniFrac distance. Similar to unweighted UniFrac, recipient samples were significantly different from donor samples (*P* < .01, F-statistic = 7.71, R vs D) but assumed a more donor-like microbiota composition after FMT (*P* > .1, F-statistic 2.1, RF vs D). R = recipient, D = donor, RF = follow-up.


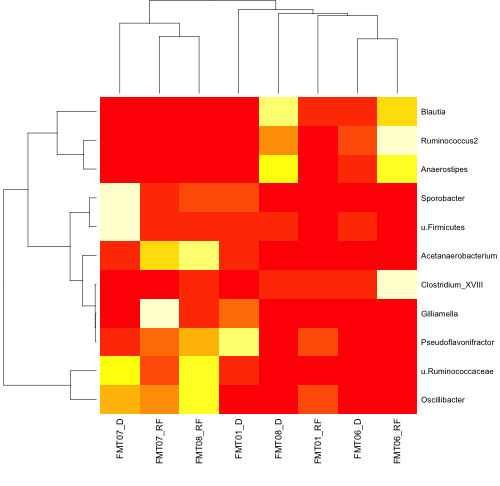

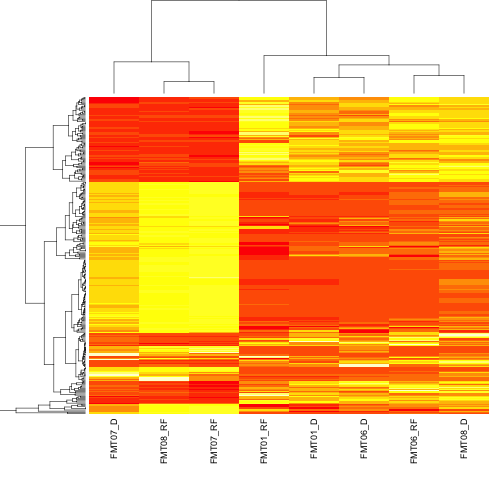


**Figure S6:** Heatmap based on metagenomic shotgun sequencing. Stratification is based on the functional and phylogenetic profiles of faecal bacterial communities which is similar. **Left:** Column dendrogram based on Spearman distance and Ward-linkage clustering. **Right:** Heatmap based on 16S-amplicon sequencing. Column dendrogram based on Unweighted UniFrac distance and Ward-linkage clustering. Shown are the bacterial genera with significant differences between the two sample groups defined by the two main branches of the dendrogram
